# Supplementary figures and images for: Reciprocity in ambiguous situations: Default psychological strategies underlying ambiguity resolution in moral decision-making
Source: PLoS One. 2024 Apr 4;19(4):e0300886. doi: 10.1371/journal.pone.0300886 (PMC10994327; doi:10.1371/journal.pone.0300886)

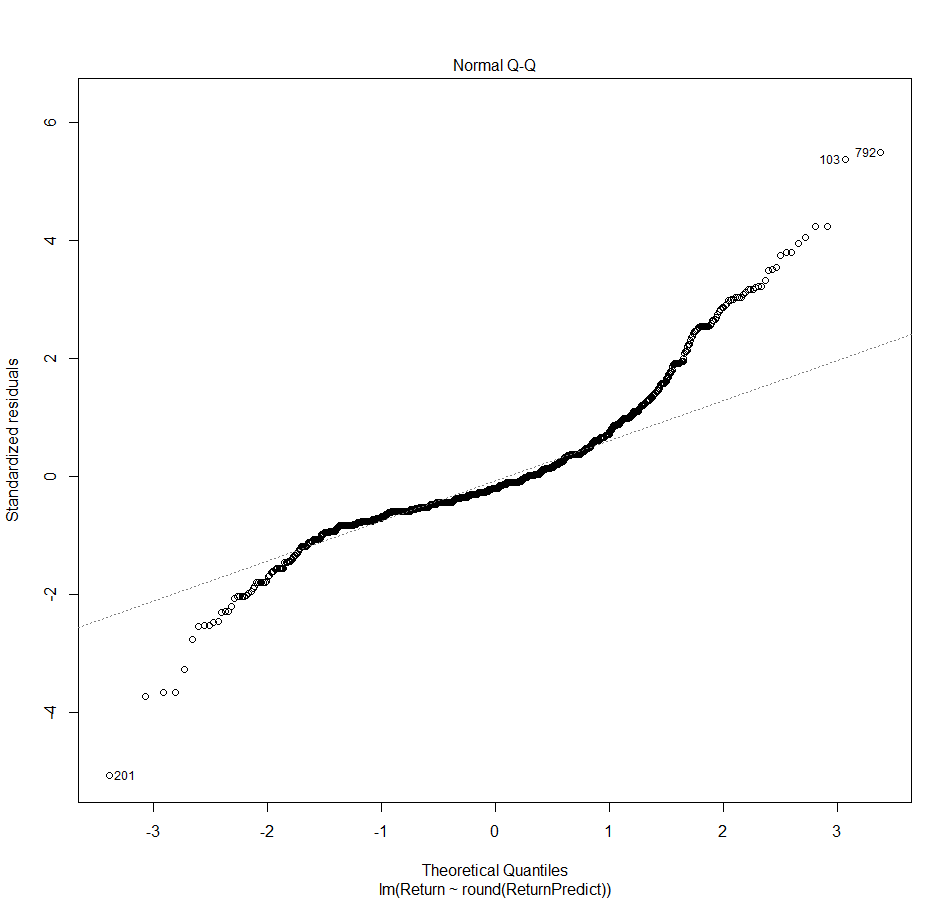

Supplement: S1 Fig — The behavioral reciprocity model provided a good fit to the data (R2 = 0.722), though model performance at higher and lower predicted values was worse than expected. (TIF) [file pone.0300886.s003.tif]
