# Supplementary material for: Reciprocity in ambiguous situations: Default psychological strategies underlying ambiguity resolution in moral decision-making
Source: PLoS One. 2024 Apr 4;19(4):e0300886. doi: 10.1371/journal.pone.0300886 (PMC10994327; doi:10.1371/journal.pone.0300886)
Supplement: Attachment — Submitted filename: RAS_Rebuttal_Final.docx [file pone.0300886.s005.docx]

Rebuttal Letter

We would like to take the opportunity to thank both reviewers for their constructive suggestions to improve our manuscript. We deeply appreciate the opportunity to submit another revision of our paper for your consideration. Below, we respond to the comments made by the reviewers and list the subsequent changes made in the paper. The page and paragraph numbers refer to those in the manuscript with track changes.

# Reviewer concerns

## Reviewer 1

Although the authors have made an effort to address most of my concerns, further refinement could enhance the manuscript's comprehensiveness and robustness. Specifically:

**1.** The manuscript would benefit from deeper discussions on behavioral trends and decision-making in ambiguous situations, with a stronger connection to psychological or moral theories. These sections, notably around pages 20 and 21, could be strengthened with additional literature citations. For example, the papers "Attributional Ambiguity Reduces Charitable Giving by Relaxing Social Norms" and "Social Game Theory: Preferences, Perceptions, and Choices" are examples of relevant literature that can enrich these discussions. While the cited papers may provide a starting point, authors should consider a broader range of literature for a richer theoretical foundation.

**Our response:**

We would like to thank the reviewer for bringing attention to this opportunity to improve our paper and, in particular, the helpful direction provided with the two recommended papers. We have now added the following paragraph to the Discussion.

*“In the context of the larger body of work on social decision-making under ambiguity, our results are somewhat anomalous: many researchers find that decision-making under ambiguity is less prosocial. Therefore, we must consider what specifically about the HETG distinguishes it from the tasks used in these previous studies. For example, in both the Public Goods Game (PGG) and Prisoner’s Dilemma (PD), wherein participants must make the decision to cooperate or defect without knowing their partner’s decision or past behavior, it has been consistently reported that participants cooperate less often than they defect (Gächter et al., 2017; Flood & Drescher, 1950; Zelmer, 2003). In a version of the PGG wherein the group must cooperate to reach a threshold of contributions to the community pot or otherwise lose all their money, participants cooperate more; however when ambiguity is introduced about what this threshold is, participants cooperate much less (Dannenberg, Lange, & Sturm, 2014). In this regard, it is possible that the specific ambiguity which is present in the HETG has a diverging effect because behavioral distrust is incentive-incompatible. In other words, participants in the HETG know that, regardless of any beliefs that they might have about how trusting investors are in general, their partners will invest more if they trust the participant more. Indeed, previous research on the PD suggests that perceptions of morality are insensitive to outcomes (Krueger et al., 2020). In his light the results from the HETG may therefore be attributed to a general aversion to behaving in what may be regarded as an immoral way simply because the context of the HETG does not offer sufficient cover to morally justify behaving selfishly. Concordantly, Tho Pesch and Dana (2024) find that charitable giving decreases when participants must make a choice about which charity to donate to before making the giving decision, rather than just making only the giving decision. The obfuscation of the charitable choice by simply providing attributes for comparison has been referred to as attributional responsibility (Snyder et al., 1979). In regard to the HETG, the lack of attributional ambiguity – or the inability to, perhaps, attribute ostensibly selfish behavior to anything other than a response to distrust, which was unmerited based on their previous experience, may be responsible for this atypical pattern of behavior.”* (page 21, paragraph 1)

**2.** Figures 1 and 2 should be improved for better legibility. This includes enhancing font size and image clarity. Additionally, the description of Figure 1 and the table in Appendix B requires more detailed explanation to ensure that readers can understand the experimental design and results without ambiguity. The manuscript currently has two appendices labeled "Appendix B" which could lead to confusion and should be rectified.

**Our response:**

We thank the reviewer for bringing attention these discrepancies. With regard to the image quality of Figures 1 and 2, we would like to reiterate that the pdf printout of the files does indeed make these images appear pixelated, since it significantly magnifies these images in order to present them at the size of the a page. However, when downloading the images one can see that the images are of substantially better quality when viewed at their original size. With regard to the text size of Figure 1, we believe that it is large enough for most to read while being small enough to keep this already large image from taking up more space on a page. Concerning the text size of Figure 2, we agree with the reviewer and have therefore made a new version of this image which is larger so as to improve legibility while preserving the proportions of each screen.

Concerning the ordering of the Appendices, we would like to express our appreciation for the reviewer’s attention to detail. During the submission of our revision, we included the previously uploaded Figures so that the reviewers could more easily reference which Appendices were being removed – however, this has clearly led to some confusion which is our fault. The only Appendix which remains and contains an image is Appendix C – showing the Q-Q plot of the behavioral reciprocity model. The former Appendix B was removed in the previous revision, which we acknowledged in point 25 of our response to your previous review: *‘We have removed what was formerly Appendix B: since the grouping of the correct parameter space was not used in our results, we see no reason to have included the a priori specified one.’* We further address this point below in our response to point 4.

**3.** The limitations section should reflect the use of a student population and suggest the need for a more diverse sample in future studies.

**Our response:**

We agree, and have now added a new paragraph outlining the limitations of using a student population, as well as the limitations associated with our use of a WEIRD population more generally.

*“A further limitation of the current study is the population which the sample was drawn from. Since our sample was comprised nearly exclusively of University students, the conclusions we can draw from our data are inherently constrained by the fact that we have sampled a portion of the adult population which is rather young, often of a relatively high SES, and share a similar educational background. For instance, respondents’ propensity to use an accuracy-oriented approach to resolve ambiguity may not be characteristic of a broader population who may be less idealistic and who, being generally less privileged, may be more self-interested. In a similar regard, the current study was conducted on a WEIRD population: WEIRD populations – which are Westernized, Educated, Industrialized, Rich, and Democratic – comprise a disproportionate majority of research on human psychology (Arnett, 2008; Rad, Martingano, & Ginges, 2018). The current study falls into this pattern: convenience samples are often used in the first stages of research, but further research into human decision-making under ambiguity must be done with a more diverse sample than we utilize here.“* (page 25, paragraph 2)

**4.** For enhanced clarity it is advised that the figure currently labeled as "Appendix B," which depicts different strategies, should be more comprehensively explained within the text. Specifically, this explanation should clearly connect to the detailed description of the parameter space provided on page 15. Such integration will help readers understand the significance of the strategies in the context of the study's outcomes and the computational modeling approach the authors have employed.

**Our response:**

We agree with the reviewer that it would help to explain what the meaning of the free parameters in our computational model. However, as we have noted previously, including the plotting of the parameter space is not merited given that we do not use the computational model to group participants because the computational model is not significant. Therefore, we believe that the most appropriate place for this discussion is in the Methods section, where we introduce the ARS model (Equation 4). Therefore, we have now added the following short paragraph after Equation 4:

“*The ARS model captures individual differences via its free parameters. It captures participants who make decisions using the heuristic strategy with its ψ parameter such that high ψ values make* *it important that the trust implied by a given endowment agrees with the trust implied by the investment alone (i.e. 1 – abs(I/E) – (I/10)).* *Inversely, low ψ values make it important that the trust implied by a given endowment agrees with the participant’s default assumed trustingness – or τ. Therefore, when ψ is high, the model predicts that participants will follow the HR strategy. When ψ is low on the other hand, τ represents the strategy: values of τ close to 0 predict the LAT strategy, values of τ close to 1 predict the HAT strategy, and values of τ close to 0.5 predict the MT strategy.“* (page 16, paragraph 2)

**5.** Ensure the accuracy of the reference list, particularly for citations like Capraro et al., to maintain consistency and correctness.

**Our response:**

Thank you for pointing out these missing references. It seems that when performing the final step of proof-reading, we neglected to ensure that the new references had been included in the Reference List. We apologize profusely for allowing this oversight to happen. The missing references and new references are now included in the new submission.

## Reviewer 2

The authors have addressed all my comments. I have only noticed that many references are missing from the new reference list. I suggest the authors to make sure that there is a matching between in-text references and bibliography.

**Our response:**

We would like to thank the reviewer for pointing out these missing references. As mentioned above, we simply neglected to ensure that the new references had been included in the reference list. We would like to express our apologies for allowing this oversight to occur. The missing and new references are now included in the new submission.

# Editor concerns

Please review your reference list to ensure that it is complete and correct. If you have cited papers that have been retracted, please include the rationale for doing so in the manuscript text, or remove these references and replace them with relevant current references. Any changes to the reference list should be mentioned in the rebuttal letter that accompanies your revised manuscript. If you need to cite a retracted article, indicate the article’s retracted status in the References list and also include a citation and full reference for the retraction notice.

**Our response:**

We thank the editor for bringing attention to accuracy of our reference list. Using our reference manager, Zotero, we have ensured that no references are made to retracted articles. Our reference article is now updated to include all citations of works in the manuscript and any further changes to our reference list are explicitly mentioned above. We apologize for this oversight and would like to express our gratitude for the opportunity to submit a further revision of our manuscript.
